# Intratumoral microbiome impacts immune infiltrates in tumor microenvironment and predicts prognosis in esophageal squamous cell carcinoma patients

**DOI:** 10.3389/fcimb.2023.1165790

**Published:** 2023-04-27

**Authors:** Shuyue Zhang, Shuishen Zhang, Xiaofan Ma, Jing Zhan, Chuqing Pan, Huizhong Zhang, Xiuying Xie, Jing Wen, Xuan Xie

**Affiliations:** ^1^ Guangdong Provincial Key Laboratory of Malignant Tumor Epigenetics and Gene Regulation, Sun Yat-sen Memorial Hospital, Sun Yat-sen University, Guangzhou, China; ^2^ Department of Thoracic Surgery, Sun Yat-sen Memorial Hospital, Sun Yat-sen University, Guangzhou, China; ^3^ Department of Thoracic Surgery, The First Affiliated Hospital, Sun Yat-Sen University, Guangzhou, China; ^4^ Department of Cardiothoracic Surgery, the First Affiliated Hospital, Guangdong Pharmaceutical University, Guangzhou, China; ^5^ Department of Intern Medicine, Zhuhai People's Hospital, Zhuhai, China; ^6^ State Key Laboratory of Oncology in South China, Collaborative Innovation Center for Cancer Medicine, Sun Yat-sen University Cancer Center, Guangzhou, China; ^7^ Guangdong Esophageal Cancer Institute, Guangzhou, China

**Keywords:** esophageal squamous cell carcinoma, intratumoral microbiome, *Lactobacillus*, prognosis, tumor microenvironment

## Abstract

**Background:**

Different intratumoral microbiotaexist in different tumors and play a crucial function in carcinogenesis. However, whether they impact clinical outcomes in esophageal squamous cell carcinoma (ESCC) and their mechanism remain unclear.

**Methods:**

16S rDNA amplicon sequencing was performed on surgically resected samples from 98 ESCC patients to analyze intratumoral microbiome abundance and composition. Multiplex fluorescent immunohistochemistry staining was used to profile the phenotypes of immune infiltrates in the tumor microenvironment (TME).

**Results:**

Patients with higher intratumoral Shannon index had significantly worse surgical outcomes. When patients were divided into short-term survivors and long-term survivors based on the median survival time, both intratumoral alpha-diversity and beta-diversity were found to be significantly inconsistent, and the relative abundance of *Lactobacillus* and *Leptotrichia* emerged as the two microorganisms that probably influenced the survival of ESCC patients. Only *Lactobacillus* in ESCC was validated to significantly worsen patients’ prognoses and to be positively correlated with the Shannon index. Multivariate analysis revealed that the intratumoral Shannon index, the relative abundance of *Lactobacillus*, and the pathologic tumor–node–metastasis (pTNM) stage were independently associated with patients’ overall survival. Furthermore, the relative abundance of both *Lactobacillus* and Shannon index was positively correlated with the proportions of PD-L1^+^ epithelial cells (ECs) and tumor-associated macrophages (TAMs). The Shannon index was negatively correlated with the proportions of natural killer (NK) cells in the TME.

**Conclusions:**

A high abundance of intratumoral *Lactobacillus* and bacterial alpha-diversity was associated with the formation of the immunosuppressive TME and predicted poor long-term survival in ESCC patients.

## 1 Introduction

Esophageal cancer remains the seventh most commonly diagnosed type of cancer and the sixth leading cause of cancer deaths globally. Of them, esophageal squamous cell carcinoma (ESCC) accounts for approximately 90% of the overall incidence (Sung et al., 2021). Although some progress has been made in surgery, chemotherapy, and radiotherapy in recent years, esophageal cancer remains a malignancy with a high degree of fatality, and its overall 5-year survival rate remains at approximately 20% (Watanabe et al., 2020). Most previous studies on esophageal cancer were focused on the changes of tumor cells, where only few of which could be transformed into clinical applications.

Tumors, including ESCC, have increasingly been recognized as organisms whose complex components contain a repertoire of recruited immune cells that contribute to the tumor microenvironment (TME) along with cancer cells. These immune cells are highly specialized, transcriptionally dynamic, and extremely heterogeneous in regard to their phenotypes and functions and have been implicated in each step of tumor development and related to prognoses of tumor patients. Recently, many studies have shown that the subtypes of TME could be new potential biomarkers for prognostic and therapeutic prediction in multiple tumors (Bagaev et al., 2021). Our previous study also showed that the infiltration of intraepithelial PD-L1^+^ tumor-associated macrophages (TAMs), memory T cells (Tmems), regulatory T cells (Tregs), and stromal granzyme B^+^ activated cytotoxic T cells (aCTLs) had clinical significance and could be used as potential biomarkers to predict prognosis in ESCC patients (Pan et al., 2021).

However, factors that regulate the TME have yet to be clearly clarified. Many factors including genes, metabolites, cytokines, bacteria, and cell interactions were proven to be somehow related to TME formation. For example, many differentially expressed genes (DEGs) were significantly related to the recruitment of various types of infiltrating immune cells (Feng et al., 2021). Oxidoreductases such as tryptophan 2,3-dioxygenase 2 (TDO2) could also cause an immunosuppressive microenvironment in ESCC by directing the polarization of M2 macrophages and promoting tumor progression (Zhao et al., 2021).

Gut microbiota are another factor that might influence the immune microenvironment. *Fusobacterium nucleatum* could promote the development of colonic neoplasia by downregulating antitumor T cell-mediated adaptive immunity (Mima et al., 2015). In murine models of colorectal cancer and melanoma, *Lactobacillus rhamnosus GG* augmented the antitumor activity of anti-PD-1 immunotherapy by increasing tumor-infiltrating dendritic cells (DCs) and T cells (Si et al., 2022). Microflora was also found to be able to colonize intratumorally because of the permissive immune-protected environment in TME, where bacteria could easily escape from host immune defenses and proliferate (Heymann et al., 2021). A recent study found that each tumor type possessed a distinct microbiome composition, and intratumoral bacteria were mostly intracellular including cancer and immune cells (Nejman et al., 2020). In a previous study, researchers found that intratumoral microbiota could modulate chemokine levels and affect CD8^+^ T-cell infiltration in the TME, consequently influencing the survival of patients with cutaneous melanoma (Zhu et al., 2021). The intratumoral microbial metabolite trimethylamine N-oxide could also enhance CD8^+^ T cell-mediated antitumor immunity in triple-negative breast cancer (Wang et al., 2022). As for ESCC, intratumoral *F. nucleatum* or *Porphyromonas gingivalis* infection was reported to be associated with a worse ESCC prognosis and reduced efficacy of chemotherapy (Yamamura et al., 2016; Gao et al., 2021a; Liu et al., 2021). However, the specific role of intratumoral microbiome diversity and composition, as well as its relationship with the TME of ESCC, remains unclear.

Here, we examined the relationship between the intratumoral microbiome and the infiltration of immune cells in the TME among 98 resected ESCC specimens. For the first time, we reported that the relative abundance of *Lactobacillus* and microbiome diversity by the Shannon index might play a role in the formation of the immunosuppressive microenvironment and served as important biomarkers for predicting the prognoses of patients with ESCC.

## 2 Methods

### 2.1 Study group

Patients with ESCC who underwent radical esophagectomy at the Department of Thoracic Oncology of Sun Yat-sen University Cancer Center from July 2003 to August 2012 were enrolled. Inclusion criteria were as follows: complete follow-up data, histologic proof of thoracic ESCC, and complete surgical resection (R0). Exclusion criteria were as follows: patients reporting the use of antibiotics or micro-ecologics for at least 2 months prior to tissue collection, other coexisting malignant tumors, preoperative neoadjuvant therapy, biotherapy, systemic inflammatory disease, or a history of gastrointestinal surgery. All the tumorous and paired non-tumorous tissues (NTs) were collected in sterile conditions immediately after tumor resection and stored at −80°C in the tumor tissue bank. NTs were derived from normal esophageal mucosal tissues located more than 3 cm away from the edge of the tumor. All cases were pathologically staged according to the Eighth Edition American Joint Committee on Cancer (AJCC) tumor–node–metastasis (TNM) staging system. The patients were followed up every 3 months in the first year, every 6 months for the next 2 years, and then annually. Overall survival (OS) was defined as the time from surgery to death, censoring patients who were still alive at the last follow-up. All the participants provided informed consent and signed the consent form of the Human Subject Institutional Review Committee. The study was approved by the Ethics Committee of Sun Yat-sen University Cancer Center (ethical approval number: B2022-070-01).

### 2.2 DNA extraction, amplification, and sequencing

DNA extraction was performed using the cetyltrimethylammonium bromide (CTAB) method. DNA concentration and purity were monitored on 1% agarose gel. DNA was diluted to 1 ng/μl using sterile water according to the concentration. The 16S rDNA V4 region was amplified using the specific primer 515F-806R with the barcode. NEB Next^®^ Ultra^™^ DNA Library Prep Kit for Illumina (NEB, Ipswich, MA, USA) was used to generate sequencing libraries and add an indexing code following recommendations by the manufacturer. The library quality was evaluated using Agilent Bioanalyzer 2100 system and the Qubit^®^ 2.0 Fluorometer (Thermo Scientific, Waltham, MA, USA). Finally, the library was sequenced on the Illumina HiSeq platform, and the paired-end reading of 250 bp was generated.

### 2.3 Sequence processing, taxonomic classification, and data analysis

Paired-end reads from the original DNA fragments were merged using FLASH, which was designed to merge paired-end reads when there were overlaps between reads 1 and 2 (Magoč and Salzberg, 2011). Paired-end reads were assigned to each sample according to unique barcodes. The Quantitative Insights into Microbial Ecology (QIIME) software package was used to analyze the sequences, and in-house Perl scripts were used to analyze alpha-diversity (within samples) and beta-diversity (among samples) (Caporaso et al., 2010). First, reads were filtered by QIIME quality filters. Then, sequences with ≥97% similarity were classified into the same operational taxonomic units (OTUs). A representative sequence was picked for each OTU, and the Ribosomal Database Project (RDP) classifier was used to annotate taxonomic information for each representative sequence (Wang et al., 2007). In order to compute alpha-diversity, the OTU table was rarified, and two metrics were calculated: OTUs and Shannon index. The OTUs functioned as an indicator of the number of tested microorganism species. The Shannon index was used to evaluate the heterogeneity of microbiota. The higher Shannon index indicates the higher alpha-diversity of the bacterial community. QIIME calculated unweighted UniFrac and Bray–Curtis distance, which were phylogenetic measures of beta-diversity. Unweighted UniFrac considered whether some bacteria with the phylogenetic relationship exist in the microbiota, without considering their abundance. However, Bray–Curtis only takes into account the relative abundance of the microbiome without considering the phylogenetic relationship. We used them for principal coordinate analysis (PCoA), which was performed to acquire principal coordinates for the visualization of sophisticated and multidimensional data. When the raw counts have been normalized to an OTU table of relative abundance, taxa of the same type were summarized at the phylum, class, order, family, and genus levels. At one level of classification, the relative abundance of a microorganism was calculated as the number of tags corresponding to the microorganism divided by the total number of tags in the sample. Student’s *t*-test was used to identify significantly different species at the phylum and genus levels. The detection of discriminant bacterial species was performed using linear discriminant analysis(LDA)of effect size (LEfSe). The LDA score indicated the effect size of each OTU, and OTUs with an LDA score >3.0 were defined as differentially abundant OTUs. Metastats was used to test the microorganism abundance between groups to get the *p*-value, and the false discovery rate (FDR) error control method (Benjamini and Hochberg false discovery rate) was used to correct the *p*-value into the Adj. *p*-value by multiple hypothesis tests. Then, microorganisms with significant differences were screened according to the *p*-value or Adj. *p*-value. Finally, the differential intratumoral microbiome was exhibited by Metastats complex heat map. Phylogenetic investigation of communities by reconstruction of unobserved states 2 (PICRUSt2) analysis was used to identify Kyoto Encyclopedia of Genes and Genomes (KEGG) pathways, and Spearman’s correlation analysis was used to analyze the intratumoral microbiome and KEGG pathway abundance (Douglas et al., 2020).

### 2.4 Hematoxylin and eosin staining

Briefly, 4-µm-thick sections of formalin-fixed paraffin-embedded (FFPE) were cut. After deparaffinization and rehydration, the slides were stained in hematoxylin for 3–5 min and washed in running water for 5 min. After differentiation in 1% hydrochloric acid (70% ethanol with 1% hydrochloric acid) (30 s), the slides were stained in 1% eosin Y for 10 min. Then, these slides were dehydrated in increasing concentrations of alcohol (each for 2 min) and cleared in xylene for observation. These slides were observed on a *Vectra Polaris* slide scanner (Akoya Biosciences, Marlborough, MA, USA).

### 2.5 Immunohistochemistry assays

Immunohistochemistry staining was performed in 4-µm sections of FFPE tumor tissues according to the standard method, including a deparaffinization and rehydration step. Antigens were retrieved in slight boiling citrate buffer (10 mM of sodium citrate, 0.1% Tween 20, pH 6.0) for 10 min in the microwave at low-to-medium power. The slides were treated with an appropriate amount of Endogenous Peroxidase Blocker (PV-9002, ZSGB-BIO, Beijing, China) for 10 min. After being rinsed with phosphate-buffered saline (PBS), Gram-negative bacteria were stained with lipopolysaccharide (LPS) Core (1:1,000, HBT-HM6011-20UG, Hycult, Plymouth Meeting, PA, USA) overnight at 4°C in a humid chamber followed by 20-min incubation of secondary antibody (goat anti-mouse IgG) at 37°C in a humid chamber. Diaminobenzidine (DAB) (ZLI-9017, ZSGB-BIO) was used for chromogenic detection for 10 min. Reactions were terminated by washing with water. Samples were stained with hematoxylin for 2 min, washed under running water for 3 min, placed in 1% hydrochloric acid solution for 20 s, washed under running water for 3 min, then blued in saturated lithium carbonate, washed, dehydrated through alcohols, cleared in xylene, and sealed with neutral resin. All slides were scanned on a Vectra Polaris slide scanner (Akoya Biosciences).

### 2.6 16S rRNA fluorescence *in situ* hybridization assays

Fluorescence *in situ* hybridization (FISH) was executed using *Direct Bacterial Fluorescent in Situ Hybridization Detection Kit*(D-0016, Guangzhou Exonbio Lab, Guangzhou, China) based on the manufacturer’s protocol. The *EUB338 16S rRNA gene probe*(GCTGCCTCCCGTAGGAGT, FB-0011B, Guangzhou Exonbio Lab) labeled with the fluorophore CY3 was used to detect bacterial colonization within human tissue by FISH. *NON338 probe*(CGACGGAGGGCATCCTCA, FB-0013B, Guangzhou Exonbio Lab) was used as a control for the hybridization protocol. Consecutive FFPE sections were hybridized by a probe that recognizes the 16S rRNA genes of all bacteria (yellow)and counterstained with *4′,6-diamidino-2-phenylindole*(*DAPI*) to visualize nuclei (blue), and tissues were visualized using a *Vectra Polaris* slide scanner (Akoya Biosciences).

### 2.7 Real-time polymerase chain reaction

Frozen ESCC samples were gently digested, and DNA was extracted in sterile conditions using the *TIANamp Genomic DNA Kit*(DP304-02, TIANGEN, Beijing, China). DNA was diluted to 1.25 ng/µl using sterile water according to the concentration. Real-time polymerase chain reaction (PCR) was performed with a *LightCycler^®^ 480 Instrument*(Roche Diagnostics, Basel, Switzerland). Briefly, amplification was performed on *384-well reaction plates* in a 10-µl final volume containing 4 µl of template DNA, 5 µl of *SYBR Green PCR Master Mix*(A25742, Biosharp), and 0.5 µl each of forward and reverse primers specific for all bacteria (F: GTGCTGCACGGCTGTCGTCA, R: A CGTCATCCACACCTTCCTC) (Shimauchi et al., 2008). Thermal cycling conditions were as follows: 95°C for 10 min, 45 cycles at 95°C for 10 s, 60°C for 20 s, and 72°C for 30 s. The C_T_ value was the cycle in which a statistically significant increase in fluorescence intensity was first detected in association with a logarithmic increase in the PCR product. The detection system constructed an amplification curve by the C_T_ value and fluorescence intensity, which meant that bacteria were detected in each sample.

### 2.8 Multiplex fluorescent immunohistochemistry staining

Tissue microarrays (TMAs) were constructed with FFPE blocks of archived tumor specimens of the recruited 98 ESCC patients. Two 1-mm cores from representative areas of each tumor sample were punched and arrayed onto a recipient paraffin block. Tissue sections (4 µm thick) obtained from TMA blocks were subjected to multiplex fluorescent immunohistochemistry (mfIHC) staining using the *PANO Multiplex IHC kit*(0004100100, Panovue, Beijing, China) to examine specific cell markers including CD11c (ab52632, Abcam, Cambridge, UK), CD45RO (55618, Cell Signaling, Danvers, MA, USA), CD68 (ZM0060, ZSGB-Bio), panCK (4545, Cell Signaling), and PD-L1 (13684, Cell Signaling) in panel A and CD4 (BX50023, BioLynx, Brockville, ON, Canada), CD8A (70306, Cell Signaling), CD56 (3576, Cell Signaling), FoxP3 (320202, BioLegend, San Diego, CA, USA), and granzyme B (ab4059, Abcam) in panel B. Different primary antibodies were sequentially applied, followed by horseradish peroxidase-conjugated secondary antibody incubation and Tyramide signal amplification (TSA). After each TSA operation, the slides were microwave heat-treated. Nuclei were stained with 4′,6′-diamidino-2-phenylindole (DAPI, D9524, Sigma-Aldrich, St. Louis, MO, USA) after all the human antigens have been labeled (Pan et al., 2021).

### 2.9 Multispectral imaging and image analysis

The way we phenotyped cells in mfIHC staining TMAs was described in detail in our previous study (Pan et al., 2021). In brief, imaging was performed using a Vectra Multispectral Imaging System (PerkinElmer, Waltham, MA, USA). One image per core was randomly captured at ×200 magnification. Each ×200 multispectral image cube was created by combining images obtained per 10-nm emission spectrum within the range of each emission filter cube. Each image capture consisted of five filter cubes, namely DAPI (440–680 nm), FITC (520–680 nm), CY3 (570–690 nm), CY5 (670–720 nm), and Texas Red (580–700 nm). InForm Cell Analysis software (PerkinElmer) was used to batch-analyze all images obtained from available cores. Each fluorophore used single antigen staining to build a library, and multispectral images were not mixed with color-based identification. Cells were phenotyped as tumoral or normal epithelial cells (ECs) (panCK^+^), tumor-associated macrophages (CD68^+^), dendritic cells (CD11c^+^), memory T cells (CD45RO^+^), cytotoxic T cells (CD8A^+^CD4^−^CD56^−^), granzyme B^+^ activated cytotoxic T cells (granzyme B^+^CD8A^+^CD4^−^CD56^−^), helper T cells (CD4^+^FoxP3^−^CD8A^−^), regulatory T cells (CD4^+^FoxP3^+^CD8A^−^), and natural killer cells (CD56^+^), and the intensity for each marker in all compartments was recorded. For each immune population, the ratio of the cell count to the total cell count was visualized by cell segmentation for analysis. The average percentage of cell counts was calculated per patient when there were two TMA cores available. Cores were excluded if no tissue was analyzable due to tissue loss or inaccurate sampling position during processing. All imaging and analysis were performed while blinded to sample identification and clinical outcomes.

### 2.10 Statistical analysis

Statistical analyses were performed with software programs SPSS 25.0 (IBM Corporation) and GraphPad Prism (version 8.0.2). Differences in OTUs and Shannon index were tested by Student’s *t*-test. To assess the potential association between microbiome and survival data, cumulative survival probability was evaluated using the Kaplan–Meier method, and differences were compared using the log-rank test. To determine independent prognostic factors, statistically significant variables in the univariate analysis were included in a multivariate Cox proportional hazards regression analysis. Statistical analyses of bacterial abundance were performed between groups of clinicopathological factors using Student’s *t*-test or analysis of variance (ANOVA). Coefficients of Spearman’s rank correlation were computed to describe associations between microbiome data and immunohistochemical quantifications. A two-sided *p* < 0.05 was considered statistically significant.

## 3 Results

### 3.1 Patients

A total of 98 ESCC patients were included in the study, including 25 patients with paired tumors and NTs. Patient characteristics are listed in **Table 1**. There were 73 male and 25 female patients, with a median age of 59 years (range 40–88 years). Pathological staging showed a slightly higher proportion of ESCC stage I–II tumors (52%). To the last follow-up date, 56 patients died from diseases related to cancer. The OS at 5 years was 47.2%, with a median OS time of 49.9 months, ranging from 7.2 to 120.9 months.

**Table 1:** Clinicopathological characteristics of 98 ESCC patients.

Variables	Patient numbers	OTUs		Shannon index		*Lactobacillus*		*Leptotrichia*	
		Mean	*p*	Mean	*p*	Mean	*p*	Mean	*p*
Sex Male Female	73 (74.5%) 25 (25.5%)	1,469.60 1,843.40	0.204^b^	6.16 6.56	0.249^b^	0.009 0.016	0.197^b^	0.024 0.015	0.150^b^
Age (years)^a^ ≤59 >59	50 (51.0%) 48 (49.0%)	1,524.00 1,607.63	0.719^b^	6.18 6.35	0.546^b^	0.010 0.012	0.502^b^	0.023 0.019	0.522^b^
Smoking status Yes No	56 (57.1%) 42 (42.9%)	1,432.09 1,742.12	0.183^b^	6.04 6.56	0.078^b^	0.009 0.013	0.368^b^	0.022 0.020	0.718^b^
Drinking status Yes No	24 (24.5%) 74 (75.5%)	1,460.00 1,599.00	0.572^b^	6.21 6.28	0.841^b^	0.008 0.012	0.407^b^	0.019 0.022	0.640^b^
pTNM stage I-II III	51 (52.0%) 47 (48.0%)	1,562.06 1,568.11	0.979^b^	6.28 6.24	0.891^b^	0.012 0.010	0.554^b^	0.029 0.013	**0.006** ^b^
Invasion depth T1–2 T3–4	24 (24.5%) 74 (75.5%)	1,626.54 1,544.99	0.779^b^	6.47 6.19	0.403^b^	0.013 0.010	0.590^b^	0.017 0.023	0.246^b^
Tumor differentiation^c^ Well Moderate Poor	19 (19.4%) 50 (51.0%) 29 (29.6%)	1,434.68 1,712.74 1,395.52	0.424^c^	6.19 6.34 6.18	0.873^c^	0.017 0.020 0.019	0.582^c^	0.020 0.027 0.013	0.183^c^
Lymph node metastasis N+ N−	52 (53.1%) 46 (46.9%)	1,567.56 1,562.02	0.981^b^	6.21 6.32	0.705^b^	0.010 0.012	0.498^b^	0.030 0.014	**0.011** ^b^
Location^c^ Upper Middle Lower	11 (11.2%) 58 (59.2%) 29 (29.6%)	1,827.73 1,493.50 1,608.21	0.656^c^	6.64 6.15 6.34	0.544^c^	0.021 0.010 0.008	0.191^c^	0.009 0.023 0.023	0.398^c^

Note. ESCC, esophageal squamous cell carcinoma; OTUs, operational taxonomic units.

a Median was used as the cutoff value.

b Students’ t-test.

c ANOVA.

Bold values indicate statistically significant.

### 3.2 Analysis of microbiome between paired ESCC and NTs

**Figure 1:**
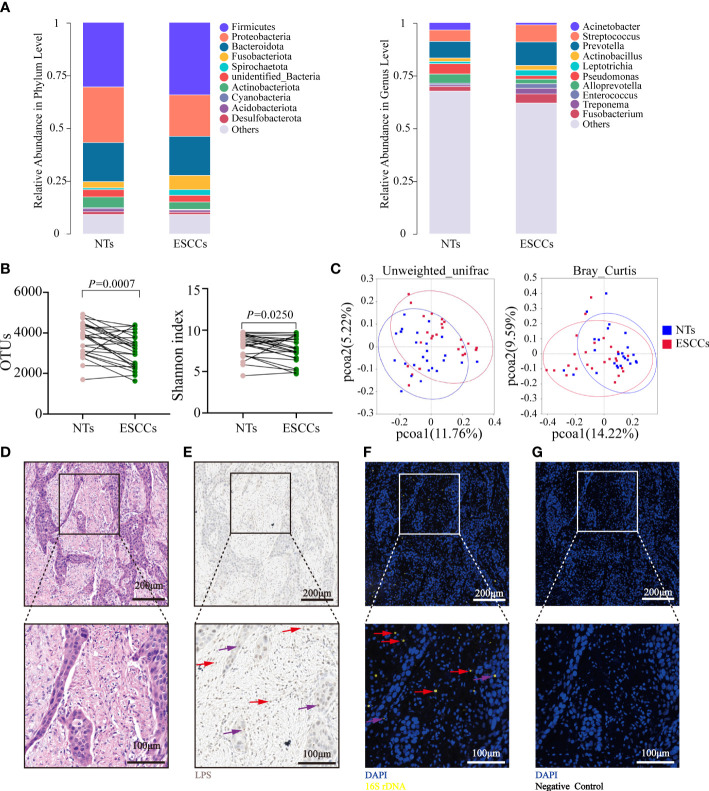
Microbiota alpha- and beta-diversity for paired ESCC and NT samples. **(A)** The top 10 relative abundance of microorganisms at the phylum and genus levels in 25 pairs of NTs and ESCC. **(B)** Shannon index and OTUs were significantly different between 25 pairs of NTs and ESCC (*p*-value by Student’s *t*-test for paired samples). **(C)** PCoA ordination plots for unweighted UniFrac and Bray–Curtis distance between 25 pairs of NT and ESCC samples (*p*-value by Wilcoxon rank sum test). **(D)** H&E staining in FFPE ESCC samples. **(E)** LPS staining in FFPE ESCC samples by immunohistochemistry using an antibacterial-LPS antibody. **(F)** FISH was used in human FFPE ESCC samples to detect bacterial 16S rRNA sequences (yellow). **(G)** Negative control of **(F)** using NON338 probe. Cell nuclei stained with DAPI (blue). The red arrows point to representative infected immune cells, while purple arrows point to representative infected tumor cells. **(D–G)** Experiments were conducted on six samples. ESCC, esophageal squamous cell carcinoma; NT, non-tumorous tissue; OTUs, operational taxonomic units; PCoA, principal coordinate analysis; FFPE, formalin-fixed paraffin-embedded; LPS, lipopolysaccharide; FISH, fluorescence *in situ* hybridization.

The microbiota characteristics of the 25 paired tumorous and NTs were analyzed. The top 5 phyla in the ESCC samples were Firmicutes, Proteobacteria, Bacteroidota, Fusobacteriota, and Actinobacteriota, while the top 5 genera were *Prevotella*, *Streptococcus*, *Fusobacterium*, *Treponema*, and *Leptotrichia*. The top 5 phyla in the NTs were Firmicutes, Proteobacteria, Bacteroidota, Actinobacteriota, and unidentified Bacteria, while the top 5 genera were *Prevotella*, *Streptococcus*, *Pseudomonas*, *Alloprevotella*, and *Acinetobacter *(**Figure 1A**). The Shannon index and OTUs were both significantly higher in NTs than in ESCC (*p* = 0.025 for the Shannon index, *p* = 0.0007 for OTUs) (**Figure 1B**), implying that microbiota in NTs were more abundant than in ESCC. Beta-diversity indicated by unweighted UniFrac and Bray–Curtis distances was significantly different between ESCC and NTs as well (*p* < 0.001), suggesting that the tumor microbial communities exhibited phylogenetic closeness within each group (**Figure 1C**). Thus, we hypothesized that the intratumoral microbiota potentially changed during the formation and development of ESCC.

Several additional experiments were conducted to confirm the presence and location of intratumoral bacteria in ESCC in six patients with matched FFPE and frozen samples. Bacterial LPS was detected in all the samples using IHC (**Figures 1D, E**), while 16S rDNA RT-PCR confirmed the existence of bacterial DNA in the corresponding frozen samples (**Supplementary Figure 1A**). The FISH analyses of bacterial 16S rRNA demonstrated that intratumoral bacteria were mostly located in the cytoplasm of tumor cells and immune cells (**Figures 1F, G**).

### 3.3 Association of intratumoral microbiome alpha-diversity with ESCC patient survival

**Figure 2:**
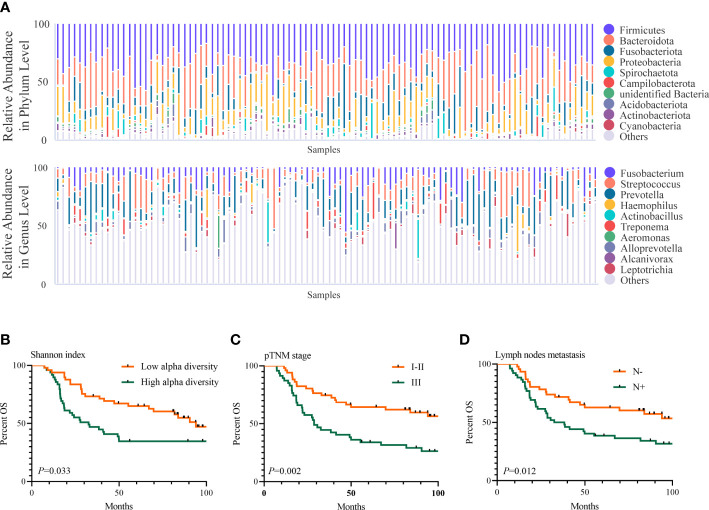
Intratumoral microbiome features in ESCC and its association with prognosis of ESCC.**(A)** The top 10 relative abundance of microorganisms at the phylum and genus levels in 98 ESCC samples. **(B–D)** Shannon index **(B)**, pTNM stage **(C)**, and lymph node metastasis **(D)** were related to OS of 98 ESCC patients (*p*-value by the log-rank test). ESCC, esophageal squamous cell carcinoma; OS, overall survival.

We further assayed the intratumoral microbiota of 98 consecutively resected ESCC samples. Based on the species accumulation boxplot and rarefaction curve (**Supplementary Figures 1B, C**), we found that the curve tended to be flat, indicating these samples were sufficient for data analysis. The top 5 phyla in the ESCC samples were Firmicutes, Bacteroidota, Fusobacteriota, Proteobacteria, and Spirochaetota, while the top 5 genera in them were *Prevotella*, *Streptococcus*, *Fusobacterium*, *Alloprevotella*, and *Treponema *(**Figure 2A**). The Shannon index ranged from 3.338 to 9.272, with a median of 6.354. The OTUs ranged from 277 to 4,350, with a median of 1,051. Neither OTUs nor the Shannon index were found to be significantly different between groups for sex, age, smoking status, drinking status, pathologic TNM (pTNM) stage, invasion depth, tumor differentiation, lymph node metastasis, and location of the tumor (**Table 1**). Then, we used the median of the Shannon index to stratify patients into the low- or high-diversity groups. Patients in the low-diversity group had significantly more prolonged OS than those in the high-diversity group (*p* = 0.033). The 5-year OS was 61.2% and 32.7%, respectively (**Figure 2B**). With respect to other clinicopathological characteristics (**Table 2**), only pTNM I–II stage (*p* = 0.002) and absence of lymph node metastasis (*p* = 0.012) were found to be associated with better OS (**Figures 2C, D**).

**Table 2:** Univariate and multivariate analyses of clinicopathological factors for overall survival in ESCC.

Variables	Univariate analysis			Multivariate analysis			
	HR	95% CI	*p* ^a^	HR	95% CI		*p* ^b^
Sex (male *vs.* female)	0.710	0.384–1.316	0.236	–	–		–
Age^c^(≤59 *vs.* >59)	0.638	0.377–1.079	0.093	–	–		–
Smoking status (no *vs.* yes)	1.023	0.602–1.738	0.933	–	–		–
Drinking status (no *vs.* yes)	1.098	0.599–2.011	0.768	–	–		–
Invasion depth (T1–2 *vs.* T3–4)	0.557	0.315–0.983	0.076	–	–		–
pTNM stage (I–II *vs.* III)	0.433	0.254–0.739	**0.002**	0.372	0.214–0.646		**<0.001**
Lymph node metastasis (N− *vs.* N+)	0.502	0.297–0.848	**0.012**	–	–		–
Shannon index^c^(low *vs.* high)	0.569	0.333–0.966	**0.033**	0.573	0.333–0.987		**0.045**
*Lactobacillus* ^c^(low *vs.* high)	0.539	0.316–0.919	**0.019**	0.527	0.307–0.905		**0.020**
*Leptotrichia* ^c^(low *vs.* high)	1.675	0.986–2.845	0.052	–	–		–
Tumor differentiation							
Well	1	–	0.360	–	–		–
Moderate	0.578	0.253–1.320	0.193	–	–		–
Poor	0.996	0.558–1.777	0.989	–	–		–
Location							
Upper	1	–	0.806	–	–		–
Middle	1.347	0.554–3.277	0.511	–	–		–
Lower	1.092	0.601–1.985	0.772	–	–		–

Note. HR, hazard ratio; CI, confidence interval.

a Kaplan–Meier method, log-rank test.

b Multivariate Cox regression analysis with forward selection.

c Median was used as the cutoff value.

Bold values indicate statistically significant.

### 3.4 ESCC intratumoral microbiome is significantly different between short-term survivors and long-term survivors

**Figure 3:**
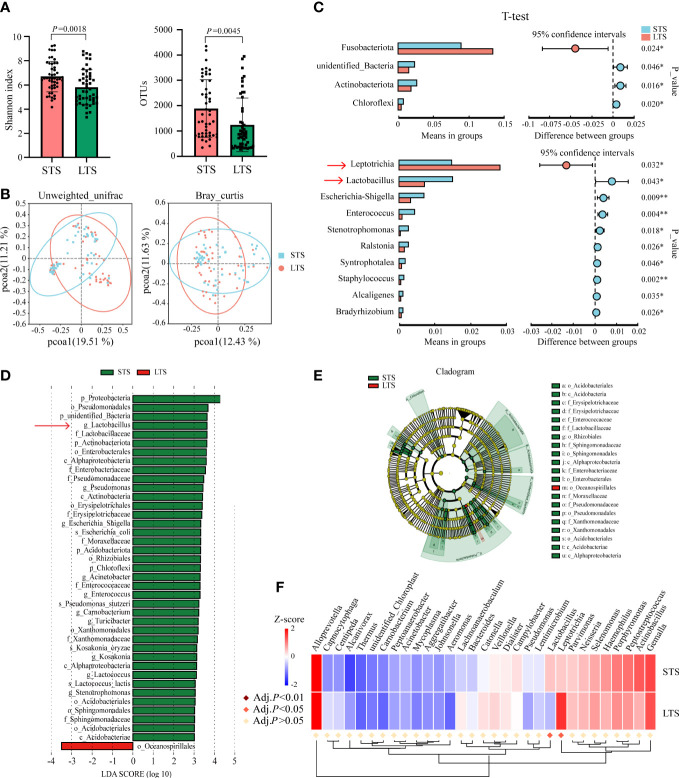
ESCC intratumoral microbiota were significantly different between STSs and LTSs. **(A)** Shannon index and OTUs were significantly different between STSs and LTSs (*p*-value by Student’s *t*-test). **(B)** PCoA ordination plots for unweighted UniFrac and Bray–Curtis distances between STSs and LTSs (*p*-value by Wilcoxon rank sum test). **(C)** Significant differences were observed between the microbiota of the STSs and LTSs at the phylum and genus levels (*p*-value by Student’s *t*-test). Only the genera whose relative abundance was more than 0.001 were exhibited. **(D)** LDA score computed from features differentially abundant between STSs and LTSs. The criteria for feature selection are log LDA score >3.0. The LDA score represents the influence extent of a microorganism for difference between STSs and LTSs. **(E)** Taxonomic cladogram of LEfSe, depicting taxonomic association between microbiota from STSs and LTSs. Each node represents a specific taxonomic type. Yellow nodes denote the taxonomic features that are not significantly different between STSs and LTSs. Red and green nodes denote the taxonomic types with more abundance in LTSs and STSs, respectively.**(F)**Metastats complex heat map of most differentially abundant features at the genus level. *Lactobacillus* and *Leptotrichia* were significantly different between STSs and LTSs (Adj. *p* < 0.05 by multiple hypothesis test). STSs, short-term survivors; LTSs, long-term survivors; ESCC, esophageal squamous cell carcinoma; OTUs, operational taxonomic units; PCoA, principal coordinate analysis; LDA, linear discriminant analysis; LEfSe, linear discriminant analysis of effect size.

To identify microorganisms that influenced the patients’ survival, we further divided patients into short-term survivors (STSs) and long-term survivors (LTSs) with the median of OS. The median OS values for STS and LTS groups were 19.1 and 95.8 months, respectively. We visualized the relative abundance of microorganisms at the phylum and genus levels in LTSs versus STSs (**Supplementary Figures 1D, E**). Then, we found that the alpha-diversity of the intratumoral microbiome was significantly higher in STSs compared with LTSs (*p* = 0.0018 for the Shannon index, *p* = 0.0045 for OTUs) (**Figure 3A**). PCoA also exhibited significant differences between STSs and LTSs calculated by unweighted UniFrac (*p* = 0.001) and Bray–Curtis (*p* < 0.001) distances, which meant that the species of OTUs were not consistent between the two groups (**Figure 3B**). Next, we examined whether there were differences in the relative abundance of each microorganism between STSs and LTSs using *t*-test, and we found that Actinobacteriota (*p* = 0.016), Chloroflexi (*p* = 0.020), and unidentified Bacteria (*p* = 0.046) were significantly higher in STSs at the phylum level. The top 5 relative abundance of genera that were significantly higher in STSs were *Lactobacillus*(*p* = 0.043), *Escherichia-Shigella*(*p* = 0.009), *Enterococcus*(*p* = 0.004), *Ralstonia*(*p* = 0.026), and *Syntrophotalea*(*p* = 0.046). However, only Fusobacteriota (*p* = 0.024) was significantly higher at the phylum level, and *Leptotrichia*(*p* = 0.032) was significantly higher at the genus level in LTSs (**Figure 3C**). LEfSe was used to conduct high-dimensional class comparisons that detected marked differences in the predominance of microbiota between STSs and LTSs. *Lactobacillus* was the most predominant microorganism at the genus level in STS tumors (**Figures 3D, E**). Survival of patients was also used as a variable to investigate whether the differential intratumoral microorganism could be segregated by Metastat complex heat map based on OTU abundance at the genus level (**Figure 3F**). Finally, combining the results of *t*-test, LEfSe, and Metastats complex heat map, *Lactobacillus* and *Leptotrichia* emerged as the representative microorganisms that potentially influenced ESCC patients’ survival. The relative abundance of *Lactobacillus* was significantly greater in STS tumors, while the relative abundance of *Leptotrichia* was significantly fewer in STS tumors (**Supplementary Figure 1F**).

### 3.5 Association of *Lactobacillus* and *Leptotrichia* abundance with patients’ clinicopathological factors

**Figure 4:**
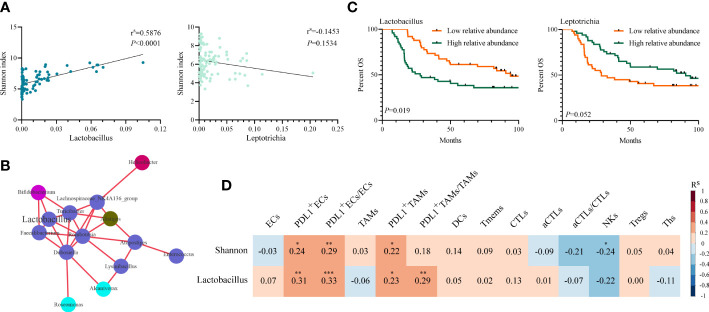
Association of intratumoral microbiome abundance with patient survival and tumor immune infiltrates. **(A)** Spearman’s correlation between Shannon index and the relative abundance of *Lactobacillus* and *Leptotrichia*(*p*-value by Spearman’s rank correlation, N = 98). **(B)** Genus level correlation network maps mainly reflected the genus (relative abundance top 100)-relatedness in tumors, correlation threshold (R^s^ ≥ 0.70 by Spearman’s rank correlation). Different colors represent different phyla, and relative abundances at genus level are directly proportional to the sizes of the circles. **(C)** Kaplan–Meier analysis with the log-rank test for OS probability based on *Lactobacillus* and *Leptotrichia* in 98 ESCC patients. **(D)** Spearman’s correlation between Shannon index or *Lactobacillus* and the percent of tumor immune cells in 98 samples (*p*-value by Spearman’s rank correlation). ^*^ *p* < 0.05. ^**^ *p* < 0.01. ^***^ *p* < 0.001. OS, overall survival; ESCC, esophageal squamous cell carcinoma.

The relative abundances of *Lactobacillus* and *Leptotrichia* were compared in groups of clinicopathological factors as shown in **Table 1**. *Leptotrichia* was present at a significantly higher abundance in the N− group than in the N+ group (0.030 *vs.* 0.014, *p* = 0.011) and in the pTNM I-II group than in the pTNM III group (0.029 *vs.* 0.013, *p* = 0.006) (**Supplementary Figure 1G**). For the other factors including sex, age, smoking status, drinking status, invasion depth, tumor differentiation, and location of the tumor, neither *Lactobacillus* nor *Leptotrichia* abundances were significantly different. When we correlated the relative abundance of *Lactobacillus* or *Leptotrichia* with the Shannon index, we found that only *Lactobacillus* abundance had a significant positive correlation with the Shannon index (*p* < 0.001, **Figure 4A**). Meanwhile, the relative abundance of *Lactobacillus* was positively related to the other intratumoral bacteria, including *Bifidobacterium*, *Turicibacter*, *Faecalibacterium*, *Romboutsia*, and *Dubosiella* (**Figure 4B**), indicating that *Lactobacillus* was one of the dominant bacteria playing an important role in maintaining the diversity of the microbiome structure in the TME. We stratified patients into low versus high categories based on the median relative abundance of *Lactobacillus* and *Leptotrichia*, respectively. Survival analyses revealed that ESCC patients with lower *Lactobacillus* abundance had a significantly better outcome (94.03 *vs.* 27.79 months, low *vs.* high, *p* = 0.019), but *Leptotrichia* abundance was irrelevant to the prognosis of ESCC patients (28.68 *vs.* 90.55 months, low *vs.* high, *p* = 0.052) (**Figure 4C**). Multivariate analysis revealed that *Lactobacillus* abundance [hazard ratio (HR) = 0.527, 95% confidence interval (CI): 0.307–0.905, *p* = 0.020], Shannon index (HR = 0.573, 95% CI: 0.333–0.987, *p* = 0.045), and pTNM stage (HR = 0.372 95% CI: 0.214–0.646, *p* < 0.001) were independently associated with OS (**Table 2**).

### 3.6 Prediction of intratumoral microbiome functions

The PICRUSt2 was carried out to predict the metagenomes from the 16S rDNA amplicon sequencing data, which were further used to identify the correlations between microbiome and KEGG pathways. A series of metabolism-related pathways including amino acid, lipid, carbohydrate, and energy, and pathways about infectious diseases and cancers were significantly positively correlated with both the Shannon index and the abundance of *Lactobacillus*(*p* < 0.05, **Supplementary Figure 1H**).

### 3.7 The relationships of ESCC intratumoral microbiome and tumor immune infiltrates

We speculated that the intratumoral microbiome might affect tumor progression through the regulation of their TME. A novel multiplex immunolabeling protocol with Opal fluorophores was used to evaluate the immune microenvironment by staining TMA sections for different cell markers in these 98 ESCC samples.

**Figure 5:**
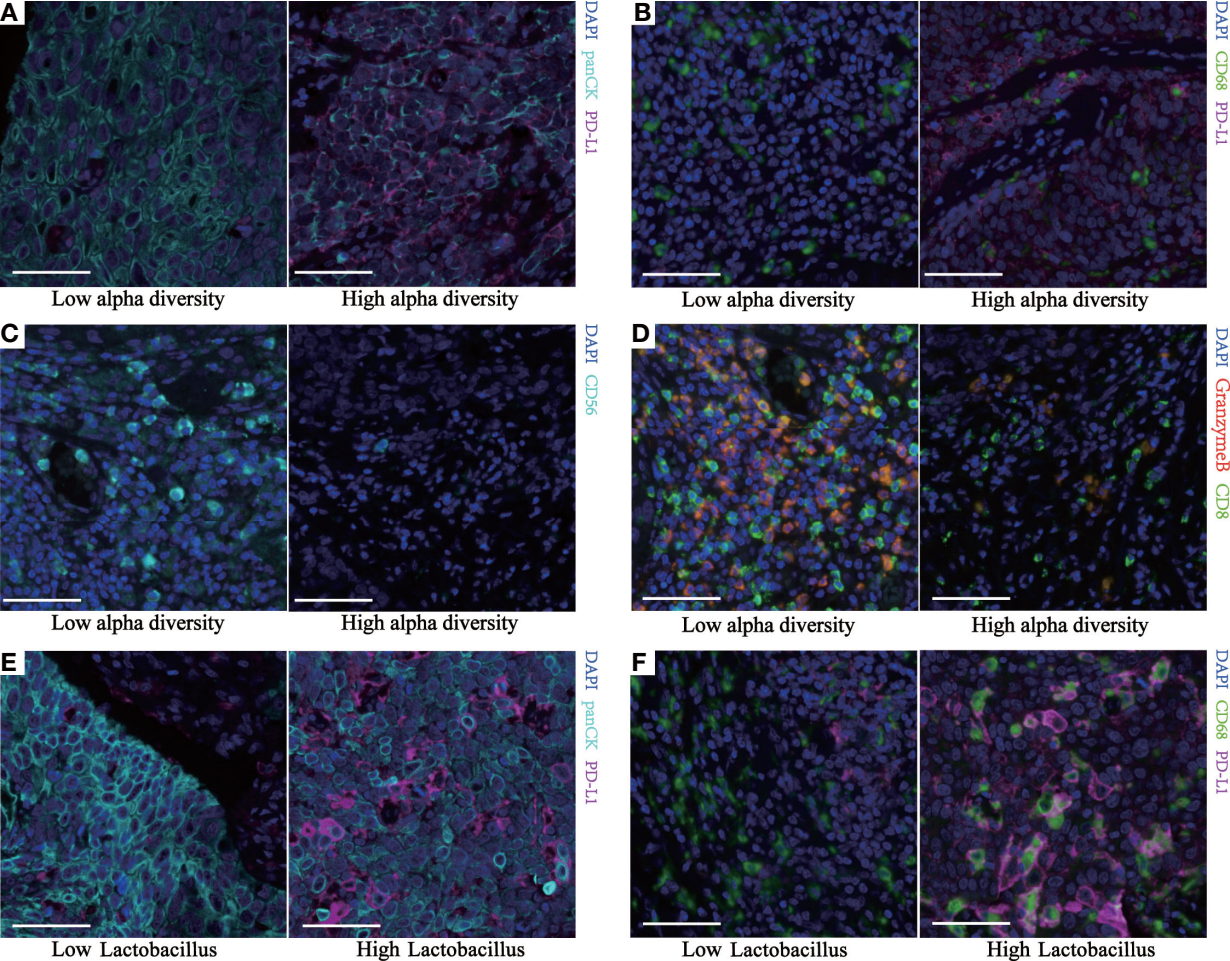
Multiplex fluorescent immunohistochemistry of human ESCC identifies different immune subpopulations. Representative images from two continuous TMA cores (**Supplementary Figure 2**) of tumor tissue for each group. Cell markers of the core are outlined in panel **(A)** (green, CD68; cyan, panCK; purple, PD-L1; blue, DAPI). Cell markers of the core are outlined in panel **(B)** (green, CD8; cyan, CD56; red, granzyme B; blue, DAPI). **(A, E)** Representative images of the different PDL1^+^ECs between two groups. **(B, F)** Representative images of the different PDL1^+^TAMs between two groups. **(C)** Representative images of the different NK cells between the two groups. **(D)** Representative images of the different aCTLs/CTLs between the two groups. All scale bars equal 50 μm. ESCC, esophageal squamous cell carcinoma; TMA, tissue microarray; ECs, epithelial cells; TAMs, tumor-associated macrophages; NK, natural killer; aCTLs, activated cytotoxic T cells; CTLs, cytotoxic T cells.

Spearman’s correlation analysis demonstrated that the Shannon index was negatively correlated with the infiltration level of natural killer (NK) cells (*p* = 0.028) and positively correlated with PD-L1^+^ECs (*p* = 0.018), PD-L1^+^TAMs (*p* = 0.033), and PD-L1^+^ECs/ECs (*p* = 0.004). Though not statistically significant, the Shannon index was still negatively associated with aCTLs/CTLs (*p* = 0.063) and positively correlated with PD-L1^+^TAMs/TAMs (*p* = 0.077) (**Figures 4D**, **5A–D**). We also found a significantly positive correlation between PD-L1^+^ECs (*p* = 0.002), PD-L1^+^TAMs (*p* = 0.020), PD-L1^+^ECs/ECs (*p* = 0.001), and PD-L1^+^TAMs/TAMs (*p* = 0.004) and the relative abundance of *Lactobacillus* in ESCC patients (**Figures 4D**, **5E, F**). The other immune infiltrates including DCs, memory T cells, Ths, and Tregs were not correlated with the Shannon index and *Lactobacillus*. These findings suggest that the intratumoral microbiome composition and the relative abundance of *Lactobacillus* might engage in the formation of the tumor immunosuppressive microenvironment and consequently promote tumor progression. Representative images displaying different levels of the Shannon index and *Lactobacillus* are shown in **Supplementary Figure 2**.

## 4 Discussion

It has been proven that the gut microbiome can remotely affect the biological behavior of tumors through blood circulation, bacterial metabolites, and enterohepatic circulation (Ma et al., 2018; Cullin et al., 2021; He et al., 2021). Since the first identification of intratumoral bacteria in solid tumors, the tumor microbiome has been the center of interest in the field of cancer research. The individual tumor could correspond to a unique microbiome, composed of different bacterial communities (Nejman et al., 2020). By characterizing the microbiota inhabiting each tumor, it is possible to learn more about the universal carcinogenic processes driving tumor progression (Lee, 2021). Here, for the first time, we reported a significant correlation between the Shannon index, signifying intratumoral microbiome alpha-diversity, and the outcome of ESCC patients.

The difference in microflora composition between tumors and their corresponding non-tumor tissue has been explored previously. These studies revealed that tumor tissue tended to have microbial depletion, including lung cancer (Kovaleva et al., 2020), liver cancer (Liu et al., 2022), and gastric cancer (Ferreira et al., 2018). In this study, we profiled the microbial alterations in the 25 pairs of tumor and normal tissue of ESCC patients using analyses based on 16S rDNA Amplicon Sequencing. Our results revealed a significantly lower microbial alpha-diversity in ESCC tumor tissue, similar to the previous studies, which also demonstrated that ESCC tumor tissue tended to have microbial depletions in comparison with physiological normal esophageal epithelium in the healthy population (Li et al., 2020; Jiang et al., 2021; Li et al., 2021). These results concluded that the microbiome was reconstituted in ESCC tissues. Although we confirmed the presence of bacteria in ESCC tissue, whether these alterations played a role in the occurrence and development of ESCC has not been elaborated thoroughly. Regarding breast cancer, Wang et al. found that tumors with different immune microenvironments were colonized by significantly different genera of commensal bacteria, indicating a different outcome and therapeutic response (Wang et al., 2022). Fu et al. found that the depletion of intratumoral bacteria could significantly reduce breast tumor metastases to the lung (Fu et al., 2022). In a genetically engineered mouse model, it has been proven that germ-free or antibiotic-treated mice were significantly protected from lung cancer development (Jin et al., 2019). Riquelme et al. reported that multiple bacteria were colonized in pancreatic adenocarcinoma (PDAC), and their combinatorial diversity in tumors could correlate to improved T-cell activation and serve as a prognostic predictor (Riquelme et al., 2019). In this study, using the Shannon index as the representative indicator of the richness of overall intratumoral microbiota, we expanded the microbiome sequencing on 98 consecutively resected ESCC samples and found that patients with a higher richness of intratumoral microflora would have a more detrimental prognosis, confirming the critical role of the intratumoral microbiome in ESCC development.

Next, we divided patients into STS and LTS groups to detect marked differences in the predominance of bacterial communities. Interestingly, *Lactobacillus* emerged as the only representative microorganism that significantly worsened the survival of ESCC patients. *Lactobacillus* has long been thought of as a probiotic that can prevent and treat a variety of chronic inflammatory diseases (Chou et al., 2020). However, whether cancer patients could benefit from its supplementation is still controversial. Bell et al. found that *Lactobacillus reuteri* and its metabolite reuterin could effectively restrict the growth of colon tumors *in vitro* and *in vivo*(Bell et al., 2022). However, in another study by Hezaveh et al., the researchers found that the removal of *Lactobacillus* by administration of antibiotic ampicillin would significantly inhibit PDAC proliferation in mice through increasing infiltration of aCTLs in the TME (Hezaveh et al., 2022). Additionally, Li et al. found that *Lactobacillus* could be a common biomarker in patients with high-grade dysplasia of the esophagus (Li et al., 2021). Wang et al. found that the relative abundance of *Lactobacillus* was negatively correlated with tumor stage, but it failed to be an independent prognostic factor in esophageal cancer patients (Wang et al., 2021). The possible reason for the conflicting results might be the small sample size and inconsistent tumor pathology (40 ESCC and 20 esophageal adenocarcinomas) in their studies. Recently, the intratumoral microbiota of ESCC and esophageal adenocarcinoma have been proven to be completely different (Baba et al., 2017). Interestingly, we also found that *Lactobacillus* abundance was positively correlated with the Shannon index. *Lactobacillus* was able to promote intestinal microbiome abundance and diversity in animals and human beings (Karlsson et al., 2010; Linninge et al., 2019; Shi et al., 2020; Xu et al., 2021). Therefore, we speculated that even if *Lactobacillus* was not the critical microorganism that directly impacted ESCC progression, it held the role of a modulator of the other bacterial richness intratumorally, together with which tumor behavior is regulated.

Gut microbiota play a pivotal role in shaping the systemic immune system (McAllister et al., 2014), but how they regulated the tumor immune microenvironment and then altered the efficacy of chemotherapy (Iida et al., 2013) and immunotherapy (Sivan et al., 2015) remained unclear. As a mucosal organ of the human body, the esophagus harbors various and abundant microorganisms, which inevitably affect intratumoral microbiota in ESCC patients (Liu et al., 2019). Esophageal mucosal barrier damage caused by the process of ESCC development may lead to the intrusion of opportunistic bacteria, while the chemotactic gradient of necrotic cellular debris in tumors may also attract microorganisms to invade the TME (Xie et al., 2022). Therefore, we hypothesized that intratumoral microbiota might be the bridge between digestive tract microbiota and the tumor immune microenvironment, influencing esophageal carcinogenesis. In this study, we used mfIHC to characterize the TME of ESCC and explored its associations with intratumoral microbiota. We demonstrated for the first time in human ESCC patients that the Shannon index was related to the formation of an immunosuppressive microenvironment, depicted by the upregulated PD-L1 expression on ECs and TAMs, and reduced infiltration of NK cells and aCTLs. Similarly, Pushalkar et al. found that the upregulation of intratumoral microbiome diversity in PDAC was associated with reduced infiltration of Ths and CTLs and increased immunosuppressive myeloid-derived suppressor cells and M2-TAMs (Pushalkar et al., 2018). In the lungs of antibiotic-aerosolized mice, a reduction in bacterial diversity was associated with enhanced T cell and NK cell activation and reduced regulatory T cells, which paralleled a significant reduction of melanoma B16 lung metastases (Le Noci et al., 2018). Our previous study revealed that the immunosuppressive microenvironment would herald a worse prognosis in ESCC patients (Pan et al., 2021). Therefore, we speculated that intratumoral microbiota might influence patients’ outcomes through modulation of TME formation. Commensal bacteria can regulate the immune system through metabolic pathways. Some strains of bacteria enable the metabolization of sugars to produce butyrate and other short-chain fatty acids, which can modulate the activity of neutrophils, macrophages, DCs, and Treg (Arpaia et al., 2013; Chang et al., 2014) and consequently promote colonic tumorigenesis (Belcheva et al., 2014). A recent study by Zhang et al. found that intratumoral Lachnospiraceae family bacteria could degrade lyso-glycerophospholipids to maintain the immune surveillance of CD8^+^ T cells and to protect against colorectal carcinogenesis (Zhang et al., 2023). Our results also revealed the correlation between the intratumoral microbiome and a series of metabolism-related pathways. However, further research is still needed to reveal the regulatory mechanism of intratumoral microbiota on the TME in cancers located in the upper digestive tract including ESCC.

PD-L1/PD-1 axis served an important role in limiting T-cell activation in the TME, resulting in tumor cells escaping immunological surveillance. It has been shown that esophageal cancer patients with PD-L1 overexpression had a significantly worse surgical outcome (Yagi et al., 2019). Moreover, PD-L1 overexpression could also be a reliable predictive biomarker for the therapeutic efficacy of immune checkpoint inhibitors. In the KEYNOTE-180 trial, participants with high PD-L1 expression would benefit more from PD-L1/PD-1 inhibitors with a higher OS rate (Shah et al., 2019). In our study, the abundance of *Lactobacillus* in the tumor tissue of patients with ESCC could impact local microbiome alpha-diversity and increase the expression of PD-L1 in ECs and TAMs. Therefore, we expected to develop ESCC therapeutics *via* manipulation of the intratumoral microbiota in the future. In the literature, *Lactobacillus* could promote immune cell activation and amplify the antitumor activity when combined with anti-PD1 immunotherapy (Si et al., 2022). Ablation of gut microbiota would significantly reduce the richness of the microbial community and weakened the efficacy of immunotherapy in multiple tumors (Pinato et al., 2019). A recent study in colorectal cancer has proved that *F. nucleatum* could induce PD-L1 expression by activating STING signaling and increase the accumulation of IFN-γ^+^CD8^+^ tumor-infiltrating lymphocytes, subsequently augmenting tumor sensitivity to PD-L1 blockade (Gao et al., 2021b).

There are some limitations in our study. First, we did not further clarify *Lactobacillus* at the species level; different species of *Lactobacillus* might have different effects on the immune microenvironment. Second, subsequent experiments are needed to determine and verify the causal relationship between the intratumoral microbiome and TME, as well as molecular mechanisms involved in the metabolism pathway. In addition, co-staining between *Lactobacillus* and immune cells is necessary to confirm the location of *Lactobacillus* in the TME, and their clearer function has to be studied in the future.

In conclusion, we demonstrated for the first time that high levels of *Lactobacillus* and bacterial alpha-diversity in tumors might influence the TME constitution, leading to a poor long-term surgical outcome in ESCC patients. Affordable and convenient detection of *Lactobacillus* abundance might be found through the Shannon index, serving as a supplemental biomarker to assess the surgical outcome of ESCC patients. In the future, specifying *Lactobacillus* family bacteria and using them as probiotics to adjust the ecology of the intratumoral microbiome might benefit the prevention and treatment of esophageal carcinogenesis.

## Data availability statement

The datasets presented in this study can be found in online repositories. The names of the repository/repositories and accession number(s) can be found below: Genome Sequence Archive (CRA007712).

## Ethics statement

The study was approved by the Ethics Committee of Sun Yat-sen University Cancer Center (Ethical approval number: B2022-070-01). The patients/participants provided their written informed consent to participate in this study.

## Author contributions

The authors gratefully acknowledge the contribution of all investigators who participated in this study. SYZ: writing—original draft, visualization, formal analysis, and software. SSZ: investigation, data curation, and supervision. XM: formal analysis and investigation. JZ: resources and methodology. CP: data curation, resources, and visualization. HZ: supervision and resources. XYX: investigation, resources, and data curation. JW: methodology, writing—review and editing, project administration, and funding acquisition. XX: conceptualization, methodology, writing—original draft, data curation, and writing—review and editing. All authors contributed to the article and approved the submitted version.

## Supplementary material

Supplementary Figure 1
**(A)** Representative real-time PCR plot showed that the bacteria 16S rDNA was amplified in 6 ESCC frozen samples. **(B)** The species accumulation boxplot of 98 samples. **(C)** The rarefaction curve of 98 samples. **(D-E)** Bar plots of microorganism relative abundance at the phylum and genus level in STS and LTS of ESCC patients. **(F)** Plots of differentially abundant genus significantly enriched in STS or LTS patients (*P*-value by Student’s t-test). **(G)** Plots of Leptotrichia significantly different in lymph node metastasis or pTNM stage (*P*-value by Student’s t-test). **(H)** Bar plot shows Spearman correlation between Shannon index or Lactobacillus and KEGG pathways abundance using PICRUSt2 analysis in 98 samples, with a false discovery rate (FDR)-adjusted *P*-value<0.05 considered significant. *Adj.*P*<0.05. **Adj.*P*<0.01. ***Adj.*P*<0.001.

Supplementary Figure 2 Multiplex fluorescent immunohistochemistry of Representative TMA cores for different groups. **(A)** The panel **(A)** of two different alpha-diversity groups. Solid wireframes were corresponding to **Figures 5A**; dash wireframes were corresponding to **Figures 5B**. **(B)** The panel **(B)** of two different alpha-diversity groups. Solid wireframes were corresponding to **Figures 5C**; dash wireframes were corresponding to **Figures 5D**. **(C)** The panel A of two different Lactobacillus abundance groups. Solid wireframes were corresponding to **Figures 5E**; dash wireframes were corresponding to **Figures 5F**. **(D)** The panel **(B)** of two different Lactobacillus abundance groups. Cell markers of the core outlined in panel A (yellow, CD11c; orange, CD45RO; green, CD68; cyan, panCK; purple, PD-L1 and blue, DAPI). Cell markers of the core outlined in panel B (yellow, CD4; green, CD8; cyan, CD56; orange, FoxP3; red, granzyme B and blue, DAPI). All scale bars equal 50 μm.
