# Supplementary figures and images for: Intratumoral microbiome impacts immune infiltrates in tumor microenvironment and predicts prognosis in esophageal squamous cell carcinoma patients
Source: Front Cell Infect Microbiol. 2023 Apr 27;13:1165790. doi: 10.3389/fcimb.2023.1165790 (PMC10174428; doi:10.3389/fcimb.2023.1165790)

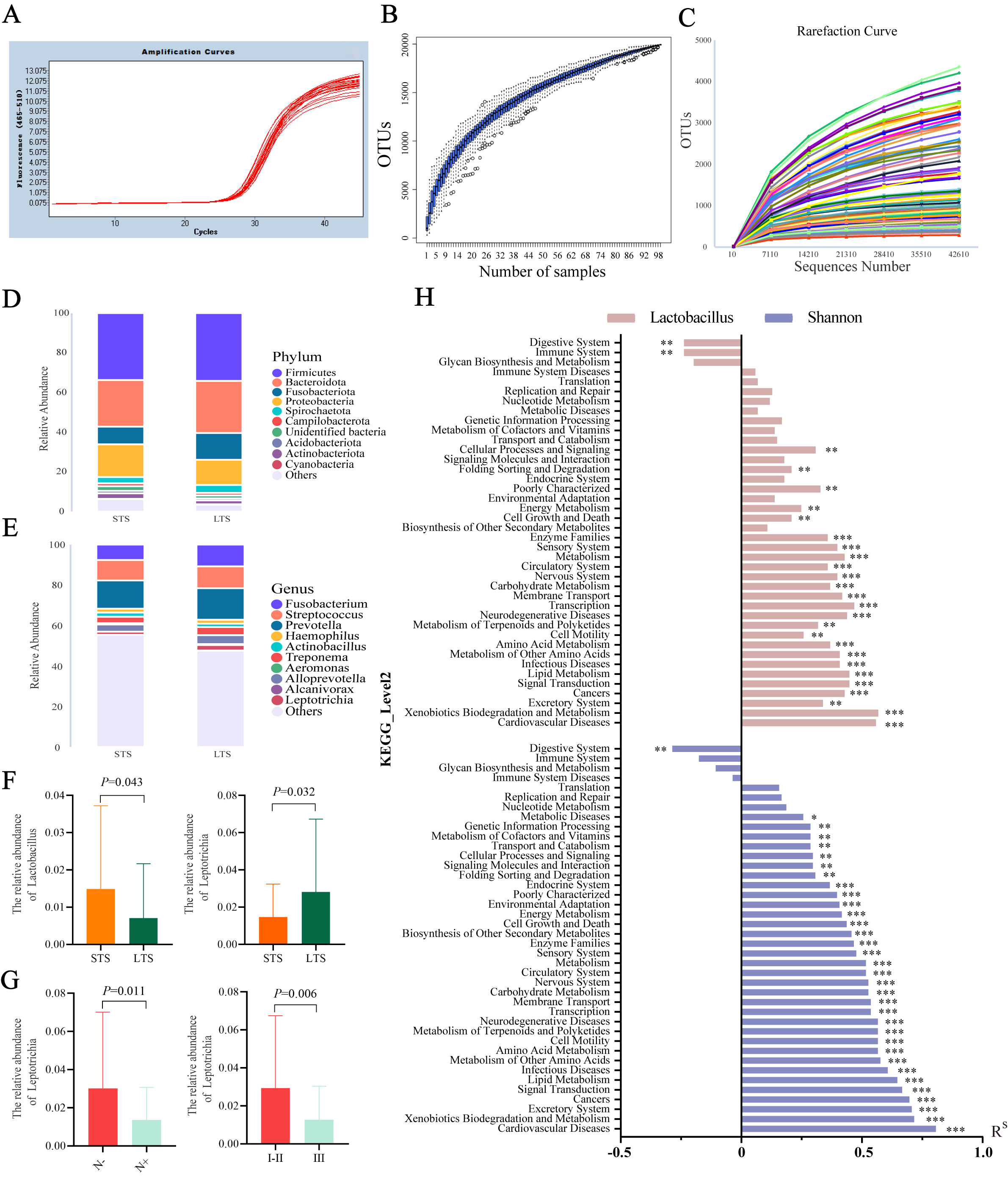

Supplement: Supplementary Figure 1 — (A) Representative real-time PCR plot showed that the bacteria 16S rDNA was amplified in 6 ESCC frozen samples. (B) The species accumulation boxplot of 98 samples. (C) The rarefaction curve of 98 samples. (D-E) Bar plots of microorganism relative abundance at the phylum and genus level in STS and LTS of ESCC patients. (F) Plots of differentially abundant genus significantly enriched in STS or LTS patients (P-value by Student’s t-test). (G) Plots of Leptotrichia significantly different in lymph node metastasis or pTNM stage (P-value by Student’s t-test). (H) Bar plot shows Spearman correlation between Shannon index or Lactobacillus and KEGG pathways abundance using PICRUSt2 analysis in 98 samples, with a false discovery rate (FDR)-adjusted P-value<0.05 considered significant. *Adj.P<0.05. **Adj.P<0.01. ***Adj.P<0.001. [file Image_1.tif]

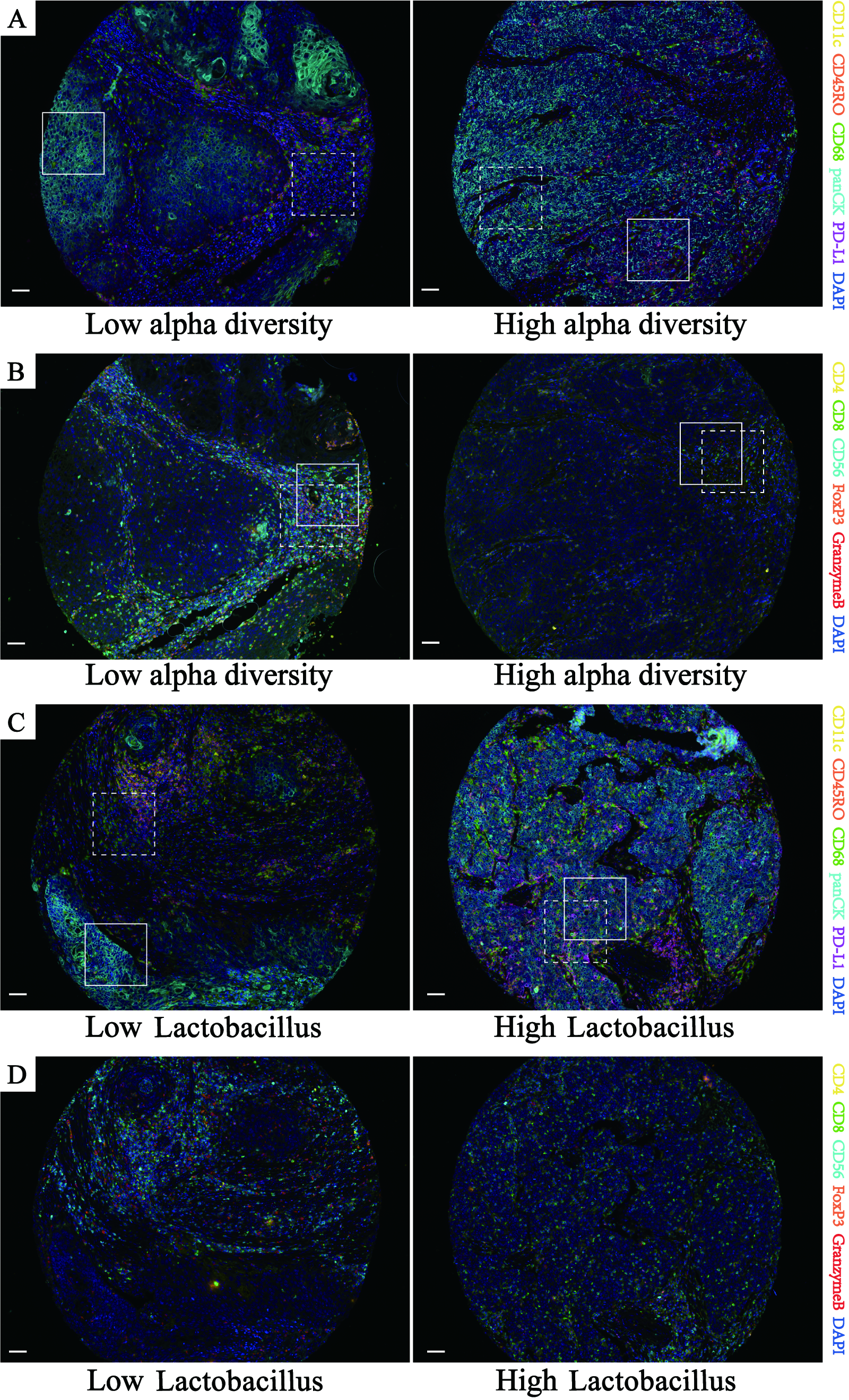

Supplement: Supplementary Figure 2 — Multiplex fluorescent immunohistochemistry of Representative TMA cores for different groups. (A) The panel (A) of two different alpha-diversity groups. Solid wireframes were corresponding to Figures 5A ; dash wireframes were corresponding to Figures 5B . (B) The panel (B) of two different alpha-diversity groups. Solid wireframes were corresponding to Figures 5C ; dash wireframes were corresponding to Figures 5D . (C) The panel A of two different Lactobacillus abundance groups. Solid wireframes were corresponding to Figures 5E ; dash wireframes were corresponding to Figures 5F . (D) The panel (B) of two different Lactobacillus abundance groups. Cell markers of the core outlined in panel A (yellow, CD11c; orange, CD45RO; green, CD68; cyan, panCK; purple, PD-L1 and blue, DAPI). Cell markers of the core outlined in panel B (yellow, CD4; green, CD8; cyan, CD56; orange, FoxP3; red, granzyme B and blue, DAPI). All scale bars equal 50 μm. [file Image_2.tif]
